# Selenylated Imidazo[1,2*-a*]pyridine Induces Cell Senescence and Oxidative Stress in Chronic Myeloid Leukemia Cells

**DOI:** 10.3390/molecules28020893

**Published:** 2023-01-16

**Authors:** Gabriella Teles Burkner, Dhébora Albuquerque Dias, Kamylla Fernanda Souza de Souza, Anna Júlia Papa de Araújo, Denise Caroline Luiz Soares Basilio, Fernanda Tondello Jacobsen, Ana Carolina Rabello de Moraes, Saulo Euclides Silva-Filho, Marcos Filipe de Oliveira Cavalcante, Cassio Augusto de Oliveira Moraes, Sumbal Saba, Maria Lígia Rodrigues Macedo, Edgar Julian Paredes-Gamero, Jamal Rafique, Eduardo Benedetti Parisotto

**Affiliations:** 1 Pharmaceutical Sciences, Food and Nutrition College, Federal University of Mato Grosso do Sul (UFMS), Campo Grande 79070-900, Brazil; 2 Department of Biochemistry, Federal University of São Paulo, São Paulo 4044-020, Brazil; 3 Department of Clinical Analysis, Center for Health Sciences, Federal University of Santa Catarina, Florianópolis 88040-970, Brazil; 4 Institute of Chemistry (IQ), Federal University of Goiás (UFG), Goiania 74690-900, Brazil; 5 Institute of Chemistry (INQUI), Federal University of Mato Grosso do Sul (UFMS), Campo Grande 79074-460, Brazil

**Keywords:** leukemia, imidazo[1,2*-a*]pyridines, selenide, oxidative stress, senescence, chronic myeloid leukemia

## Abstract

Imidazo[1,2*-a*]pyridines (IPs) have been studied regarding drug development. The objective of this work was to evaluate the antileukemic capacity of IP derivatives by screening their ability as a pro-oxidant. IP derivatives were synthesized and oral bioavailability and toxicity were analyzed in silico. Redox screening was performed on human Kasumi, KG-1, K562, and Jurkat leukemia cells. The IP derivative and the most responsive leukemic cell were selected for cytotoxicity, cell proliferation, cell senescence, and oxidative stress assays. The predictive toxicity analysis showed a possible effect on the reproductive system, but without mutagenic, carcinogenic, or irritability effects. MRK-107 against K562 cells was the compound that showed the best redox profile. MRK-107 did not induce cell death in K562 and monocyte cells. However, this compound was able to decrease cell proliferation and increase cell senescence after 48 and 72 h. Furthermore, MRK-107 induced oxidative stress in K562 cells after 72 h, increasing lipid peroxidation and decreasing reduced glutathione (GSH) contents. This study demonstrated that MRK-107-induced senescence with the involvement of oxidative stress is a possible mechanism of action, addressing this compound as a potential antitumor drug against chronic myeloid leukemia.

## 1. Introduction

Leukemia is a set of malignant disorders that present an excess of leukocytes in the blood and/or bone marrow [1]. There is a range of hematopoietic malignancies currently subcategorized according to their morphology, immunophenotype, cytogenetic and molecular abnormalities, and clinical features [2].

Chronic myeloid leukemia (CML) is a myeloproliferative neoplasm or myeloproliferative disorder that accounts for 15% of adult leukemias [3]. Since the arrival of imatinib in the market in 2022, there has been a sharp decrease in annual mortality from 10–20% down to 1–2% [3]. Despite the success of this therapy, the prevalence of CML has increased, and it has been suggested that in 2040 the incidence rate and mortality rate will balance [4,5]. However, the cure of CML is functionally not molecular because imatinib does not act directly on the bases of CML but competes for the ATP binding site of tyrosine kinase, restoring the cell death mechanism [6]. Therefore, it is of great interest to search for medications that increase the rate of complete answers and that improve the eradication of minimal residual disease (MRD), with fewer side effects and less toxicity.

Compounds that have imidazo[1,2*-a*]pyridines (IPs) in their core structure have been widely used in medicinal chemistry and drug development. This is due to the fact that these compounds are correlated with interesting therapeutic properties, e.g., antineoplastic [7], anti-inflammatory [8], antidiabetic [9], and antimalarial effects [10]. Besides, IPs are known to be potent P3IK/mTOR inhibitors with promising kinase selectivity, inducing apoptosis and cell cycle arrest [11]. On the other hand, organoselenium compounds modulate many biological processes, including oxidative stress, overgeneration of reactive oxygen species (ROS), DNA damage, and mitochondrial dysfunction [12]. Considering the therapeutic importance of IPs and the biological relevance of organochalcogen compounds, the molecular hybridization of these two chemical structures results in some interesting pharmacological properties [13,14,15]. The properties of chalcogenylated-IPs have been related to an important strategy in drug research as novel chemotherapeutic agents, as they can increase the antineoplastic effects in less toxic and equally effective doses, taking DNA and cell death [16].

As IPs induce oxidative stress in tumor cells [13,14], we hypothesized their involvement in the antiproliferative mechanism by inducing the senescence of leukemic cells. Cell senescence is a programmed arrest of growth that prevents cell proliferation [17]. Thus, when apoptosis does not induce cancer cell death, the induction of cell senescence is an alternative for tumor suppression [18].

Thus, considering our continuous interest in the synthesis of organoselenium compounds with biological relevance and functionalization of IPs [19,20,21,22,23,24,25,26], the main goal of the present research is the quest for new drugs for the treatment of CML. Considering that oxidative stress, induction of apoptosis, and senescence are crucial processes involved in the response of cancer cells to therapy, we used IP and its chalcogen derivatives in leukemic cell lines to investigate their antitumor effects with an interest in potential chemotherapeutic activity against the CML.

## 2. Results and Discussion

The parameters of oral bioavailability and toxicity evaluated using the SwissADME software and Osiris^®^ Property Explorer, respectively, are shown in Table 1. According to this analysis, only one violation of the Lipinski rules was presented [27].

According to Lipinski and colleagues (1997), these rules are related to the molecular properties that are necessary for the studied compound to present good physicochemical characteristics such as solubility, intestinal permeability, and oral bioavailability [27]. Therefore, only the compound MRK-116 can present problems with solubility, oral bioavailability, and consequently absorption because its mLogP is higher than the ideal value of 4.15. All the other compounds showed promising oral bioavailability.

In addition, regarding toxicity, all the compounds presented a low risk for all the parameters evaluated, such as mutagenicity, tumorigenicity, irritability, and effects on reproduction. Therefore, all the compounds were considered promising in the in silico studies. Consequently, in vitro studies were initiated to evaluate the antileukemic potential of IPs.

**Figure 1:**
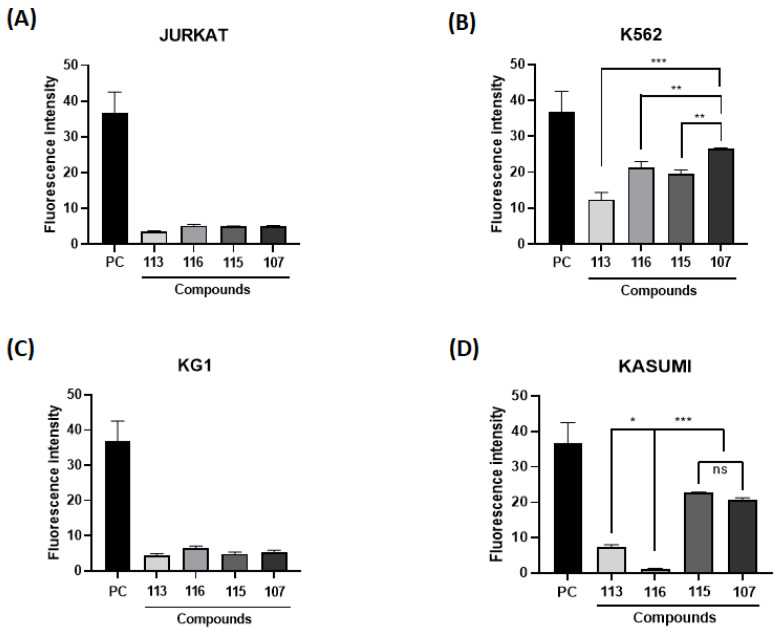
Intracellular reactive oxygen species (ROS) content. Fluorescence intensity obtained in different leukemic cells [(**A**) Jurkrat; (**B**) K562; (**C**) KG1; (**D**) Kasumi)] after treatment with imidazo[1,2*-a*]pyridines (10 µM). (*), (**), and (***) mean statistical differences for *p* < 0.05, *p* < 0.01 and *p* < 0.001, compared among the compounds, respectively. (ns): not significant.

The generation of ROS is necessary for normal cell function, but it may also be suggested in cancer therapy. Therefore, cell lines from leukemic models were incubated with imidazopyridine compounds to assess the potential of these compounds to induce intracellular ROS generation. Cells were exposed to the same concentration of 10 µM and analyzed by fluorescence. The cells used in the screening were Jurkat (acute lymphocytic leukemia—ALL), K562 (MCL), KG1 (acute myeloid leukemia—AML), and Kasumi (AML) (Figure 1).

It can be noticed in Figure 1B that the cell line K562 showed the highest fluorescence intensity, as well as the highest rate of intracellular ROS, notably the marker 107. The application of ROS in the therapeutic field of hematopoietic cancer cells has been linked to a therapeutic mechanism, as anticancer drugs induce an increase in ROS generation, leading cancer cells to apoptosis or senescence [28,29]. Thus, the K562 and MRK-107 cell lines were selected for the following assays, as MRK 107 was the IP compound that showed the best oxidative profile.

**Figure 2:**
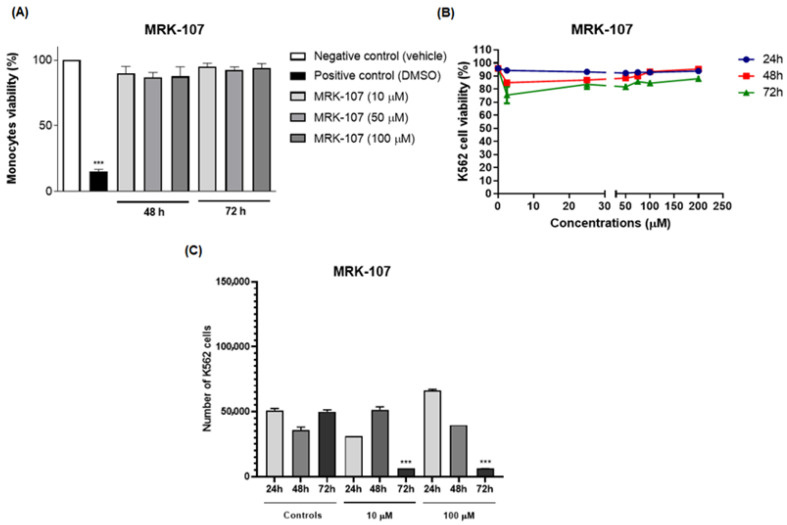
Effect of treatment with MRK-107 at different concentrations on cell viability and proliferation. (**A**) Percentage of monocyte cell viability at 48 and 72 h; (**B**) percentage of viability of K562 cells after MRK-7 treatment at 24, 48, and 72 h; (**C**) cell counts after MRK-7 treatment at 24, 48, and 72 h. (***) *p* < 0.001 compared to the control group/negative control.

In the cell viability assay, MRK-107 was tested in monocytes at different concentrations. MRK-107 at concentrations of 10, 50, and 100 µM/mL at 48 and 72 h showed no significant difference in cell viability when compared to the negative (Figure 2A). These results indicate that MRK-107 did not induce cytotoxicity at the tested concentrations.

The assay was performed to assess cell viability and evaluate the cytotoxic and proliferative effects induced by MRK 107, which could eventually lead to cell death. The cells K562 were stimulated with MRK-107 for 24, 48, and 72 h and analyzed by flow cytometry (Figure 2B). The test demonstrated that the administration of MRK-107 was not time dependent, as cell viability was not significantly affected, suggesting that cell death by apoptosis did not occur. However, the proliferation assay (Figure 2C) showed that after 72 h there was a significant reduction in cell count, demonstrating that MRK 107 induced an inhibition of cell proliferation.

The results obtained corroborated those of Santos and collaborators who, through the analysis of a compound derived from selenylated imidazopyridine, obtained a significant reduction in cell counts when compared to cells not treated with the compound; that is, they reported that imidazopyridine derivatives have an antiproliferative potency in the cells of glioblastoma [30]. Another study by Almeida and collaborators using the same imidazopyridine-derived compound demonstrated an inhibition of cell growth in 90% of breast cancer cell lines when compared to untreated controls [13]. Another study carried out by Santos and collaborators also evaluated the antiproliferative effect and the oxidative damage of selenylated IPs in hepatocarcinoma cell lines and obtained a reduction in the proliferative capacity of 80% after 72 h, that is, also corroborating that compounds derived from imidazopyridines induce a state of cell death [14]. Therefore, the study was continued to evaluate the mechanism of senescence.

**Figure 3:**
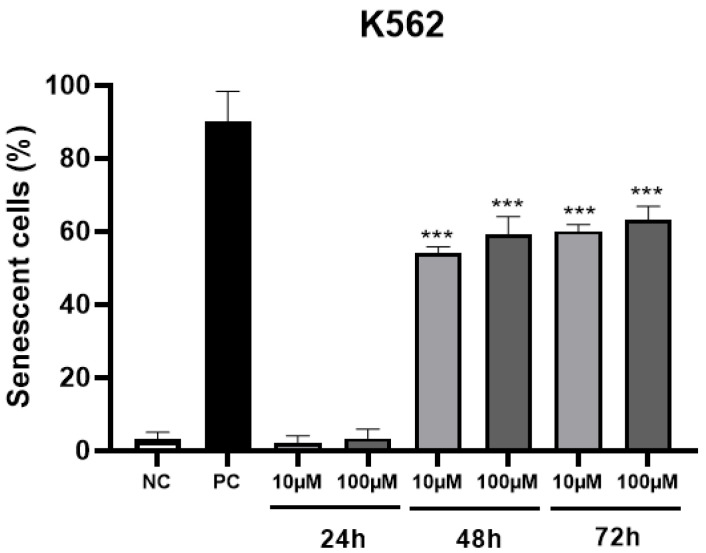
Percentage of senescent K562 cells after 24, 48, and 72 h of MRK-107 incubation (10 and 100 µM). (***) *p* < 0.001 compared to the negative control (NC). PC: positive control.

The percentage of senescent cells in 48 and 72 h (Figure 3) at two non-toxic concentrations of MRK-107 was significantly higher than 50%, which indicates that the administration of this compound is able to induce cells to enter senescence, thereby being a desired and consistent effect for chemotherapy.

It is well known that senescence is one of the tumor suppressor mechanisms, and that it causes an irreversible interruption of cell proliferation, and that this condition can be induced in response to chemotherapy [31,32,33]. Senescence is a mechanism that causes the cell cycle to stop in order to potentially inhibit cell cycle progression and consequently allow the proliferation of dysfunctional or transformed cells to occur [34,35].

The mechanisms that cause senescence are broad and include ROS overgeneration, DNA damage, and mitochondrial dysfunction [36], while the use of radiotherapy and chemotherapy drugs, for example, is known as “therapy-induced senescence” [34]. Guo and collaborators (2010) published that treatment with hydrogen peroxide (H_2_O_2_), which induces oxidative stress, resulted in 80% of mouse embryonic fibroblasts becoming senescent, which corroborates the present study since MRK 107 showed a high rate of intracellular ROS in K562 cells, as well as a significantly higher rate of senescent cells after treatment with the respective marker [37]. Accordingly, Zhong and collaborators (2019), in a study with breast cancer cells, also demonstrated that oxidative stress mediated by H_2_O_2_ treatment also caused an induction of senescence in these cells [38].

In addition, a review study on hydroxyurea (HU), an antineoplastic drug widely used in neoplastic and non-neoplastic conditions, reported that the compound is able to reduce cell proliferation as it can induce a state of cell senescence [39]. According to some related studies, HU promotes a deficiency of proteins that regulate oxidative stress, thus contributing to the elevation of ROS and, consequently, to the establishment of cellular senescence [40,41,42]. These findings are well in line with the data obtained in the present study, as they indicate that increased levels of ROS contribute to cellular senescence. In such a way, senescence may be a protective mechanism against tumor growth that prevents an uncontrolled proliferation of cancer cells or cells that contain some oncogene activation or the loss of tumor suppressor genes, corroborating some other related studies that also support senescence as a proliferation suppressor mechanism that can stop tumor growth [43,44].

**Figure 4:**
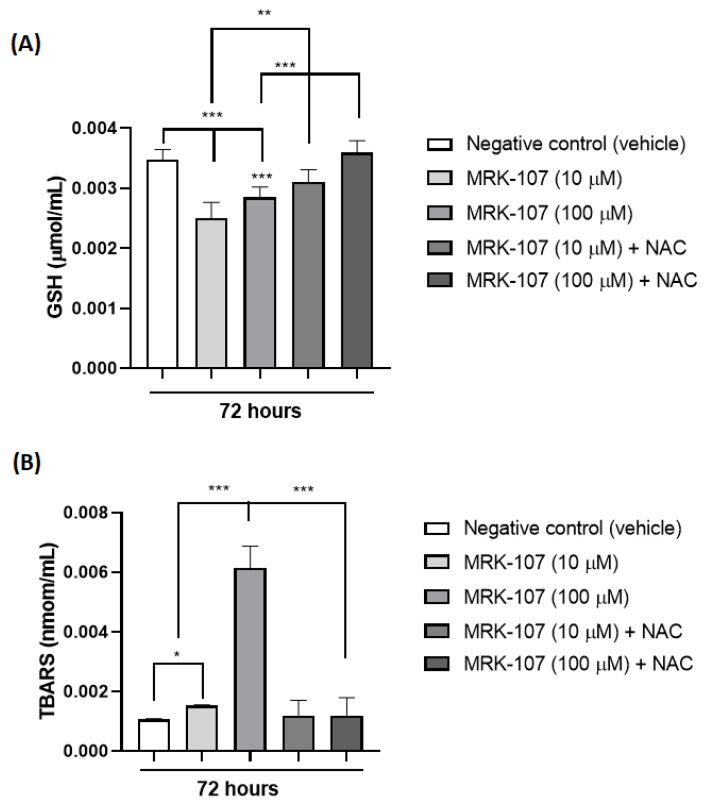
Oxidative stress markers in K562 cells (**A**) Amount of TBARS (nmol/mL) after stimulation with MRK-107 (10 um and 100 um) in 72 h. (**B**) Amount of reduced gluthatione-GSH (umol/mL) after stimulation with MRK-107 (10 uM and 100 uM) at 72 h. (*), (**), and (***) mean statistical differences for *p* < 0.05, *p* < 0.01 and *p* < 0.001, compared among the groups, respectively.

Through the analysis of TBARS and reduced glutathione (GSH) in 72 h, it was possible to observe the presence of oxidative stress after stimulation of the K562 cell with MRK-107 (Figure 4). TBARS (nmol/mL) is a product of lipid peroxidation of the cell membrane, which occurs in the presence of oxidative stress, while GSH is a naturally consumed antioxidant in the presence of oxidative stress, thus increasing TBARS (Figure 4A) and decreasing GSH (Figure 4B) indicate the presence of oxidative damage induced by MRK-107.

The studies of cell damage associated with senescence are still difficult to detect; however, several studies have studied the most toxic forms with more immediate effects, such as DNA damage. DNA breakage is considered one of the most toxic forms of cell damage, and one of its immediate consequences is cell cycle arrest, that is, senescence, as it is known that telomere shortening and chromosomal instabilities are well-established factors for senescence. In addition, studies report an increase in oxidized intracellular compounds, such as lipofuscin and carbonyl, glycated and modified proteins by lipid peroxidation in aged senescent cells [45].

These data corroborate other findings in the literature as well, in which a low level of GSH in the brain of mice was found associated with the detoxification of endogenous toxins originating through cellular lipoperoxidation processes arising from an insufficient removal of H_2_O_2_ that favors the production of oxidative stress [46]. In addition, in that same study, a higher density of senescent cells was associated with mice whose brains more exposed to oxidative stress, demonstrating that oxidative stress is an important factor that causes senescence [46].

## 3. Conclusions

The results of the present study allow us to conclude that the MRK-107 compound obtained the best oxidative profile, and the K562 cell was the most responsive cell. However, MRK-107 did not induce cell death in K562 cells or human monocytes but decreased the proliferation of K562 cells after 72 h of exposure as well as induced cell senescence at 48 and 72 h of exposure. Furthermore, the compound MRK-107 induced oxidative stress in K562 cells after 72 h of exposure.

In conclusion, this work demonstrated that the senescence induced by the compound MRK-107 has the involvement of oxidative stress as a possible mechanism of action, being a potential antitumor mechanism in the chemotherapy of cancer cells using compounds derived from IP against the CML.

## 4. Materials and Methods

### 4.1. Synthesis of Imidazo[1,2-a]pyridines and Chalcogenated Derivatives

**Figure 5:**
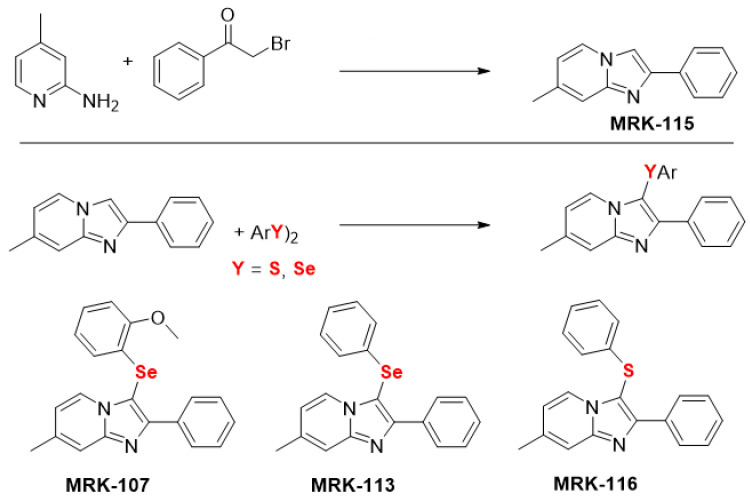
Chemical structure of the imidazo[1,2-*a*] pyridine [47] and its chalcogen derivatives [48,49,50,51], used in this study.

The starting material, imidazo[1,2*-a*]pyridine MRK-115, was synthesized by refluxing 2*-a*mino-4-methylpyridine and bromoacetophenone in ethanol for 4 h [47]. A series of chalcogenated imidazo[1,2*-a*]pyridine (MRK-107, MRK-113, MRK-116) were synthesized through C(sp2)-H bond selenylation/sulfenylation of imidazo[1,2*-a*]pyridines MRK-115 using diorganyl diselenides/disulfides (Figure 5), as previously described by us [48,49,50,51].

### 4.2. Oral Bioavailability and Toxicity of Compounds: In Silico Analysis

The oral bioavailability of the compounds was evaluated using the SwissADME software, an online tool developed by the Swiss Institute of Bioinformatics (SIB) which is available at http://www.swissadme.ch/ (accessed on 22 October 2021) that predictively evaluates the pharmacokinetics, based on the similarity to medicines and the medicinal chemical compatibility of compounds. Compounds were evaluated according to criteria established by Lipinski et al. (1997) [27] and Veber et al. (2002) [52], namely: mLogP, molecular weight (MW), number of hydrogen bond acceptors (N and O atoms), number of hydrogen bond donors (NH and OH radicals), number of rotatable bonds, and area of polar surface (TPSA).

Toxicity tests were performed using Osiris^®^ Property Explorer, a free program that, by analyzing the chemical structure of compounds, shows relevant properties of drugs and possible drugs. The properties evaluated were: mutagenicity, tumorigenicity, irritability, and negative effects on reproduction.

### 4.3. Cell Culture

Human leukemic cell lines (Kasumi-1, KG-1, K562, and Jurkat) were obtained from the American Type Culture Collection (ATCC). KG-1 cells were maintained in Iscove’s Modified Dulbecco’s Medium (IMDM) supplemented with 20% fetal bovine serum (Cultilab, Campinas, Brazil). The other lineages were maintained in Roswell Park Memorial Institute (RPMI 1640) medium supplemented with 10% fetal bovine serum. All lineages were cultured in medium containing 100 U/mL penicillin and 100 μg/mL streptomycin. The lineages were maintained in a humidified incubator containing 5% CO_2_ at 37 °C. Buffer and chemicals were purchased from Sigma*-*Aldrich (Darmstadt, Germany). The lineage cells were maintained for 4 weeks.

Peripheral blood mononuclear cells were collected by peripheral venipuncture from three male healthy volunteers (between 20 and 30 years old and unmedicated) in polypropylene tubes containing 3.8% sodium citrate (1/10, *w/v*). All human volunteers provided informed consent. Separation of mononuclear cells was performed by gradient centrifugation methods using Ficoll-Histopaque-1077 (1.077 g/cm^3^) (Sigma-Aldrich, Darmstadt, Germany). The use of human samples was approved by the local Ethical Committee of the Universidade Federal de Mato Grosso do Sul (CAAE 35853720.2.0000.0021). The cells were maintained in IMDM supplemented with 20% FBS, at the same condition as described above.

### 4.4. Redox Effect Screening: Measurement of Intracellular ROS

Initially, the screening of IP and leukemia cells was performed based on the redox effect. Then, the leukemic strain most responsive to treatments with the compounds (best oxidative profile) was selected for the following stages of the study. Intracellular ROS content was evaluated as reported by [53]. Human leukemic cell lines were incubated for 12 h with IP (10 μM), washed twice with HBSS, and then 100 μL of HBSS/well was added. After that, the cells were loaded with DCFH-DA (10 μM) in HBSS at 37 °C and incubated for 30 min. Excess DCFH-DA was removed by washing with fresh HBSS. The intensity of fluorescence was measured at 485 nm for excitation and 530 nm for emission using a Multiscan microplate reader (Thermo Fisher Scientific Oy^®^, Vantaa, Finland). The results of the fluorescence intensity of the compounds were obtained by discounting the baseline values of the cells not exposed to the compounds (negative control). A positive control with 100 µM H_2_O_2_ was performed.

### 4.5. Cytotoxicity Assay

The 2,5-diphenyl-2H-tetrazolium bromide (MTT assay) was performed as previously described [54] and using human monocytes. Briefly, monocytes were dispensed in 96-well culture plates and placed in an incubator at 37 °C, 5% CO_2_. After 24 h, MRK-107 (10, 50, and 100 μM/mL, 100 μL) was diluted in IMDM medium and added. Cells unstimulated were used as a negative control. After 48 and 72 h, the supernatant was removed and MTT (5 mg/mL) was added and incubated for 4 h. Then, the medium was removed and lysis solution (200 μL of DMSO) was added by well and homogenized for 20 min. The absorbance was read at 540 nm in a microplate reader (HumanReader HS, Wiesbaden,·Germany). The results were expressed as percentage (%) of viable cells.

### 4.6. Cell Death Assay

K562 cells were plated (10^5^ cells/mL) and stimulated with MRK-107 (0–200 μM) for 24, 48, and 72 h. After this period, the cells were washed and resuspended in the buffer solution (0.01 M HEPES, pH = 7.4, 0.14 M NaCl, and 2.5 mM CaCl_2_). The suspensions were labeled with propidium iodide (PI—1 µg/mL) (Becton Dickinson, Franklin Lakes, NJ, USA) according to the manufacturer’s instructions. The cells were incubated at room temperature for 20 min. A total of 10,000 events were collected per sample. Flow cytometry evaluation was performed on a flow cytometer. Data were analyzed using the FlowJo v10.8 Software (BD Life Sciences, Franklin Lakes, NJ, USA).

### 4.7. Cell Proliferation Assay

K562 cells were incubated with MRK-107 at concentrations of 10 and 100 μM for 24, 48, and 72 h. Cell counting was performed using a Neubauer chamber [55]. The growth constant was calculated using the logistic growth equation.

### 4.8. Senescence Assay

SA-β-galactosidase (β-Gal) activity by cytochemistry was performed to assess the induction of cellular senescence [56]. K562 cells were plated (10^5^ cells/mL) and incubated with MRK-107 (10 and 100 μM) for 24, 48, and 72 h. After, the cells were washed in PBS and incubated at 37 °C (no CO_2_) with fresh senescence-associated stain solution: 1 mg of 5-bromo-4-chloro-3-indolyl β-D-galactoside (X-Gal) per ml [stock = 2 mM MgCl_2_, 5 mM K_4_Fe(CN)_6_3H_2_O, 5mM, K_3_Fe(CN)_6_ PBS buffer, pH 7.4]. Cell staining and morphology were assessed by microscopy after 12 h (microscope: Leica^®^). The results were expressed as a percentage of senescent K562 cells. A negative control (vehicle) and a positive control (100 µM H_2_O_2_) were used.

### 4.9. Oxidative Stress Markers

Oxidative stress markers were evaluated in K562 cells treated with the MRK-107 compound (10 and 100 µM) for 72 h [57]. The cells (48 × 10^6^ per 600 μL) were homogenized in a lysis solution (cold buffer containing 20 mM sodium phosphate, pH 7.4, 150 mM NaCl, and 0.1% Triton) for the determination of lipid peroxidation. To measure the concentration of reduced glutathione (GSH), the cells were homogenized in 12% TCA (trichloroacetic acid). The determinations were performed with the supernatant after centrifugation (5000× *g* for 5 min). The N-acetyl cysteine (NAC) antioxidant was used as a free radical scavenger.

#### 4.9.1. Lipid Peroxidation Assessment

Lipid peroxidation was determined by measuring thiobarbituric acid reactive substances (TBARS), mainly malondialdehyde (MDA) [58]. Briefly, the homogenate was precipitated with 12% TCA, followed by incubation in solution (60 mM Tris–HCl, pH 7.4, 0.1 mM diethylenetriaminepentaacetic acid), and 0.73% thiobarbituric acid (TBA), at 100 °C, for 60 min. After cooling, the samples were centrifuged (5 min at 10,000× *g*) and the absorbance was measured at 535 nm. The results were expressed in nmol/mL.

#### 4.9.2. Reduced Glutathione Assay (Non-Protein Thiols)

The measurement of the reduced glutathione (GSH) content was performed in acid extracts, using the reagent DTNB (5.5′-dithiobis-2-nitrobenzoic acid) [59]. After being centrifuged at 5000× *g* for 5 min, the supernatants were added to 2.5 mM DTNB in 0.2 M sodium phosphate buffer, pH 8.0, and the formation of the yellow thiolate anion was immediately measured at 412 nm. The results were expressed in μmol/mL.

### 4.10. Statistical Analysis

The results were expressed as the mean ± standard deviation (SD). The data were analyzed using a two-way ANOVA followed by the Bonferroni or Tukey–Kramer test. Values of *p* < 0.05 were considered statistically significant. Statistical analysis was obtained from three independent experiments and was performed by GraphPad Prism software, version 8.0 (San Diego, CA, USA).

## Figures and Tables

**Table 1:** Theoretical parameters of oral bioavailability and toxicity of compounds.

Compound	MRK-107	MRK-113	MRK-115	MRK-116
mLogP	3.37	3.76	2.54	4.61
MW (g/mol)	393.34	363.31	208.26	316.42
n° of violations	0	0	0	1 (mLogP > 4.15)
Mutagenicity	-	-	-	-
Tumorigenicity	-	-	-	-
Irritability	-	-	-	-
Effects on reproduction	Yes	Yes	Yes	Yes
Absorption in the GIT	High	High	High	High

Note: MW: molecular weight; GIT: gastrointestinal tract.

## Data Availability

Not applicable.
